# Effect of continuous measurement and adjustment of endotracheal tube cuff pressure on postoperative sore throat in patients undergoing gynecological laparoscopic surgery: study protocol for a randomized controlled trial

**DOI:** 10.1186/s13063-023-07406-w

**Published:** 2023-05-27

**Authors:** Chen Wang, Xiang Yan, Chao Gao, Simeng Liu, Di Zhang, Jia Jiang, Anshi Wu

**Affiliations:** 1 Department of Anesthesiology, Beijing Chaoyang Hospital, Capital Medical University, Gongrentiyuchang South Road No. 8, Beijing, China; 2 Department of Clinical Epidemiology, Beijing Chaoyang Hospital, Capital Medical University, Beijing, China

**Keywords:** Endotracheal tube cuff pressure, Pressure transducer, Monitor, Sore throat, Postoperative complications, Randomized controlled trial

## Abstract

**Background:**

Postoperative sore throat (POST) is a common postoperative complication after endotracheal tube removal. There are still no effective preventive methods for POST. The aim of this trial is to confirm whether maintaining intraoperative cuff pressure below the tracheal capillary perfusion pressure could effectively reduce the incidence of POST among patients undergoing gynecological laparoscopic surgery.

**Methods:**

This study is a single-center, randomized, parallel-controlled, superiority trial with a 1:1 allocation ratio. Sixty patients whose age is between 18 and 65 years and scheduled for gynecological laparoscopic surgery will be randomized to the cuff pressure measurement and adjustment (CPMA) group and the only cuff pressure measurement without adjustment group (control group). The primary endpoint is the incidence of sore throat at rest within 24 h after extubation. The secondary endpoints include the incidence of cough, the incidence of hoarseness, the incidence of postoperative nausea and vomiting (PONV), POST, and pain intensity within 24 h after extubation. Blocked randomization will be conducted with a computer-generated central randomization online service. The blind method will be applied to subjects, data collectors, outcome evaluators, and statisticians. Outcome assessments will be performed at 0 h and 24 h post-extubation.

**Discussion:**

This randomized controlled study hypothesizes that cuff pressure is the primary influencing factor of POST. By continuous monitoring of endotracheal tube cuff pressure and maintaining it within the range of 18–22 mmHg compared with only continuous measurement without adjustment, it aims to prove that continuous measurement and adjustment of endotracheal tube cuff pressure could be effective in reducing the incidence of POST in gynecological laparoscopic surgery patients. The result of this study could be used as a reference for future multicenter studies to confirm the effect of cuff pressure on POST and provides a scientific theoretical basis for preventing POST to further support comfort medicine.

**Trial registration:**

Chinese Clinical Trial Registry ChiCTR2200064792. Registered on 18 October 2022. This protocol (version 1.0, 16 March 2022) was approved by the Ethics Committee of Beijing Chaoyang Hospital.

## Background

As one of the problems frequently complained about by patients, postoperative sore throat (POST) is a common postoperative complication after extubation with an incidence of 14–62% [1–3]. POST refers to the throat pain caused by irritation or damage to the mucosa or airway structures due to airway manipulation or the airway device itself. Previous systematic reviews show that factors such as female sex, high cuff pressure, and inappropriately sized tube are associated with POST [3, 4]. However, there is as yet no clear evidence to point out the main cause of POST. Therefore, the principle goal of the current researches is to identify the main influencing factor so as to effectively intervene and prevent POST.

Some studies suggest that high cuff pressure of the endotracheal tube may be a vital contributing factor [5, 6]. The ideal cuff pressure is one that does not affect the blood perfusion of tracheal mucosa, achieves no airway leakage during ventilation, and prevents ventilator-associated pneumonia (VAP) due to the aspiration of subglottic secretions. Accordingly, it is recommended to maintain cuff pressure in the range of 25–30 cmH_2_O (18–22 mmHg) after inflation, lower than the capillary perfusion pressure of the tracheal mucosa [7, 8]. At present, two methods are commonly used to monitor cuff pressure in clinical practice. Many anesthesiologists prefer the pilot balloon palpation method to estimate the cuff pressure despite the risk of endotracheal cuff overinflation [9]. Most respiratory physicians’ choice to measure cuff pressure is an endotracheal cuff pressure manometer. Although the manometer is accurate to measure cuff pressure, manual intermittent measurement and close observation are required. Each time the manometer is connected to the pilot balloon and then separated, there is a drop in cuff pressure of 2–3 cmH_2_O [10]. Moreover, a pressure transducer has been reported in studies [11–13] as a better method to measure cuff pressure than the two mentioned above.

Cuff pressure measurement by the manometer can reduce the incidence of POST in patients scheduled for gynecological laparoscopic surgery [14]. However, no studies have yet investigated the different effects of pressure transducer-guided cuff pressure measurement with or without adjustment on POST in patients undergoing gynecological laparoscopic surgery.

## Objectives

The aim of this study is to confirm whether continuous measurement of endotracheal tube cuff pressure and maintaining it at the normal value (18–22 mmHg) compared with only continuous measurement without adjustment could effectively reduce the incidence of POST in patients undergoing gynecological laparoscopic surgery.

## Methods

### Study design

This is a single-center, parallel-group, randomized controlled, superiority trial with a 1:1 allocation ratio. The time schedule of the protocol (Table 1) is based on the Standard Protocol Items: Recommendations for Interventional Trials (SPIRIT) 2013 [15].

**Table 1:** The schedule of enrollment, interventions, and assessments

	Study period			
	Enrollment	Allocation	Post-allocation (post-extubation)	
Time point	− 1 day before surgery	Day of surgery	0 h	24 h
Enrollment				
Eligibility screen	X			
Informed consent	X			
Allocation		X		
Interventions				
CPMA group		X		
Control group		X		
Assessments				
Baseline variables	X	X		
Number of patients with POST at rest			X	X
Number of patients with cough			X	X
Number of patients with hoarseness			X	X
Number of patients with PONV			X	X
Pain intensity (NRS)			X	X

### Study setting

This randomized controlled study will be performed at Beijing Chaoyang Hospital, Capital Medical University, Beijing, China.

### Study population

#### Inclusion criteria

Patients scheduled for elective gynecological laparoscopic surgery under general anesthesia will be recruited. Participants with ages between 18 and 65 years, body mass index (BMI) of 18–30 kg/m^2^, and American Society of Anesthesiologists (ASA) classes I–II must voluntarily sign a written informed consent before the intervention.

#### Exclusion criteria

Patients will not be eligible with one of the following criteria: preoperative cough, sore throat, or upper respiratory tract infection; preoperative nasogastric tube insertion; smoking history; history of nausea and vomiting; history of oropharynx and larynx surgery; or other conditions that will interfere with the benefits of patients in the trial identified by researchers.

### Interventions

No preoperative medication will be used in all patients. After entering the operating room, patients will be routinely monitored for non-invasive arterial blood pressure, electrocardiogram, pulse oxygen saturation, end-tidal carbon dioxide, and then inhale oxygen through a mask. After peripheral venous catheterization, midazolam (0.02–0.05 mg/kg), sufentanil (0.2–0.5 μg/kg), propofol (2–3 mg/kg), and rocuronium (0.6–0.8 mg/kg) will be injected intravenously during induction of general anesthesia. The anesthesiologists with at least two years of experience will select polyvinyl chloride endotracheal tubes of 7.0 mm internal diameter for video laryngoscope-guided intubation. In the cuff pressure measurement and adjustment (CPMA) group, the anesthesiologist will connect the pressure transducer to the spring-loaded one-way valve of the pilot balloon through a three-way connector and then inflate or deflate the tube cuff with a syringe connected to a tee-junction to maintain 18–22 mmHg cuff pressure intraoperatively. In the control group, the tube cuff pressure will be adjusted by anesthesiologists’ experience with pilot balloon finger palpation before the operation and will not be adjusted during the operation but will be monitored blindly on the portable monitor which screen will be fully covered. The number of tracheal intubation attempts, cricoid compression, and cough during intubation will be recorded. After successful endotracheal intubation, volume-controlled ventilation will be performed with a tidal volume of 6–8 ml/kg, a respiratory rate of 12–18 breaths per minute, an inspiratory-to-expiratory ratio of 1:2, an inspired oxygen fraction of 80%, and a flow rate of 1–2 L/min to adjust end-expiratory carbon dioxide partial pressure to 35–45 mmHg. Total intravenous anesthesia will be maintained with propofol (4–6 mg/kg/h) and remifentanil (0.1–0.25 μg/kg/min). Additional rocuronium 10–20 mg and sufentanil 5–10 μg will be administered as needed during the operation. After the establishment of carbon dioxide pneumoperitoneum, the patients in both groups will be placed in the Trendelenburg position required for gynecological laparoscopic surgery. The pneumoperitoneum pressure will be kept at 14 mmHg. Hemodynamic changes will be monitored and recorded every 5 min during the operation. Operation time and endotracheal tube indwelling time will also be recorded. After the surgery, gentle suction of oropharyngeal secretions will be performed in both groups (the wall suction pressure ranges from − 80 to − 120 mmHg). The administration of intravenous muscle relaxant antagonist (neostigmine 40 μg/kg) will be determined based on the assessment of muscle strength recovery. Once the patient is fully awake, the tidal volume has been restored to normal, and sufficient oxygen saturation can be maintained, air will be withdrawn from the cuff, and the endotracheal tube will be removed. In addition, the number of coughs before extubation will be recorded. Both groups of subjects will receive postoperative pain control via an analgesic pump with sufentanil at a rate of 2 μg/h and a total dose of 100 μg. Sore throat at rest and its severity, cough, hoarseness, nausea and vomiting, and pain score will be recorded immediately after extubation (0 h after extubation) and within 24 h after extubation. The investigators will record conditions in which enrolled subjects are deemed unfit to proceed with the study due to the following reasons: occurrence of adverse events, including severe adverse events, and other circumstances identified by the researchers as unsuitable for further experimentation. To enhance participants’ adherence with the intervention measures, researchers will employ clear and comprehensible language to communicate with patients, conduct timely follow-ups, and engage in interactive discussions. The researchers will also record timely during the intervention and follow-up process to monitor the compliance of the subjects.

### Outcomes and measurements

#### Primary outcome

The primary outcome is the incidence of sore throat at rest within 24 h after extubation. The total number of patients with sore throat at rest between two assessment time points (0 h and 24 h post-extubation) will be recorded.

#### Secondary outcomes

The secondary outcomes include the following: (1) the incidence of cough within 24 h after extubation; (2) the incidence of hoarseness within 24 h after extubation; (3) the incidence of PONV within 24 h after extubation; (4) the intensity of POST within 24 h after extubation graded on a 0–3 scale: 0 = no sore throat, 1 = mild sore throat (less pain than a cold), 2 = moderate sore throat (like a cold), and 3 = severe sore throat (more severe than a cold) [16–19]; and (5) 11-point Numerical Rating Scale (NRS) for pain intensity within 24 h after extubation: 0 = no pain and 10 = worst pain imaginable [20].

### Sample size calculation

The sample size estimation is based on the incidence of sore throat while resting within 24 h post-extubation. This superiority trial with a 1:1 allocation ratio will achieve 80% power (1 − β) and a one-sided significance level (*α*) of 0.025. In a randomized controlled study of gynecologic laparoscopic surgery [14], the incidence of POST was 31.7% (19/60) in cuff pressure adjusted to 25 cmH_2_O group (using the manometer) and 68.3% (41/60) in the control group (using palpation of the pilot balloon). Based on this study of a similar surgery, we estimate the incidence of sore throat at rest within 24 h post-extubation is 32% in the CPMA group and 68% in the control group. Then, 54 participants should be needed according to the conditions mentioned above. Eventually, a total of 60 patients (30 in each group) will be recruited so as to allow for a 10% dropout rate. The sample size was calculated using tests for two proportions in the PASS software (version 15, NCSS Corp., USA).

### Recruitment

The recruitment process will be simplified. Unnecessary application documents and requirements will be reduced. Emphasizing the significance and benefits of the study is crucial to stimulate the interest and willingness to participate among potential participants.

### Assignment of interventions

#### Randomization

Participants will be randomly assigned to either the CPMA or the control group with a 1:1 allocation on the day of surgery using Ericure, which is a computer-generated central randomization online service [21]. Blocked randomization using permuted blocks of random sizes will reduce the predictability of the allocation sequence. The allocation sequence in the central randomization system will be managed by a statistician independent of the research. Blinded study personnel who are unaware of the allocation sequence will receive a randomization code via a central randomization system on the day of surgery, following subject screening and all baseline measurements, thereby ensuring allocation concealment. The allocation sequence will be concealed without the influence of selection bias until the completion of the database analysis.

#### Blinding

As a result of the different types of intervention, neither anesthetists nor gynecologists will be blinded to the allocation. However, the patients, the data collectors, the outcome assessors, and the statistician will be masked to the information about the allocation. For example, an observer who is unaware of the allocation will assess for sore throat of the patients within 24 h after extubation. Unblinding will be performed after the completion of the database analysis. If a serious adverse event occurs in the participant, the principal investigator will log into the central randomization system for emergency unblinding.

### Data collection and management

Clinical data of the patients will be collected by trained researchers who are unaware of the group assignment. All clinical trial data will be recorded using an electronical case report form (eCRF). After the data monitoring staff verifies the accuracy and completeness of the source document, the data entry personnel will establish the database with the EpiData software (version 3.1, EpiData Association, Denmark). For any subjects who drop out of or deviate from the trial for any reason, the reasons will be recorded as thoroughly as possible. Prompt and appropriate measures will be taken to address or treat their withdrawal, ensuring completion of follow-up and reporting of all observed outcomes. After logical range checks for data values, the database with independent double data entry will be locked and backed up.

### Statistical analyses

All data will be analyzed by the SPSS software (version 26, IBM Corp., USA). Both the intention-to-treat analysis and the per-protocol analysis will be performed for the primary outcome. Subgroup analyses will be performed based on the values of the incidence of cough, hoarseness, and PONV; the intensity of POST; and NRS for pain intensity within 24 h after extubation. A two-sided significance level will be set at *P* < 0.05 for all statistical tests. The continuous variables will be expressed as a mean with standard deviation (normal distribution) or a median with interquartile range (skewed distribution). Student’s *t* test will be used to compare normally distributed continuous variables, and the Mann–Whitney *U* test will be used to compare non-normally distributed continuous variables. The categorical variables such as the incidence of sore throat, cough, hoarseness, and PONV within 24 h after extubation will be described as counts and percentages. Differences between the groups of these categorical variables will be compared with *χ*^2^ analysis or Fisher’s exact test. The missing data will be handled by multiple imputation.

### Data monitoring

Due to the short duration of this study and the minimal known safety risks, no formal data monitoring committee will be established. However, a trained auditor will monitor the progress of the study every month. The auditor must ensure that the clinical facilities at the research center will meet the requirements of the study and that the investigators will follow the protocol and accurately record the results of the study. For each inspection, the auditor will check the eCRF, the original documents, and the storage conditions of drugs and devices. The auditor will also ensure that each participant has signed informed consent.

### Safety assessments

All adverse events, including serious adverse events, observed in the clinical trial will be recorded on the eCRF. The duration of the adverse event, its severity, its relationship with the study device, and its appropriate treatment will be recorded. The patients will be followed up until remission or recovery from adverse events.

### Ethics and dissemination

#### Protocol amendments

For modifications that do not affect the rights of the participants and the evaluation of trial results, exemption applications can be made by the principal investigator and submitted to the Ethics Committee of Beijing Chaoyang Hospital for record keeping. All other modifications to the trial protocol will require approval from the Beijing Chaoyang Hospital Ethics Committee.

#### Consent

The researchers will introduce themselves and explain the purpose, procedures, and potential risks and benefits of the trial to the potential participants. The researchers will obtain participants’ written consent by providing consent forms. These forms will outline the key points of the trial and will be signed by the participants.

#### Confidentiality

Strict confidentiality measures will be implemented throughout the trial. Personal information collected from potential and enrolled participants will be treated as sensitive and confidential. It will be stored securely, with access limited to authorized personnel only.

#### Access to data

The final trial dataset will typically have restricted access and will be available to authorized individuals. The principal investigator and members of the study team directly involved in the trial will have access to the final trial dataset. These individuals will be responsible for analyzing and interpreting the data for research purposes.

#### Dissemination policy

The results will be disseminated in a peer-reviewed journal and at academic conferences.

Authorship eligibility guidelines will be established to ensure transparency and fairness in assigning authorship for clinical trial reports. The authors should have made substantial contributions to the design, conduct, interpretation, and reporting of the clinical trial. Merely providing general support or administrative assistance will not be typically considered sufficient for authorship. Before publicly providing the full protocol, participant-level dataset, and statistical code, we will ensure that the data is appropriately processed and de-identified to protect the privacy and data security of the participants.

## Discussion

POST may be disappearing within 48 h in most cases, but it is not conducive to the postoperative comfort of patients. If it is not taken seriously, patients who have retained the endotracheal tube for a long time (such as patients in intensive care units) may have more serious complications than POST, such as tracheomalacia, laryngotracheal stenosis, and tracheoesophageal fistula. This is a parallel-group, randomized controlled, superiority trial with a robust methodology to confirm whether maintaining intraoperative endotracheal tube cuff pressure below the tracheal capillary perfusion pressure could effectively reduce the incidence of POST in patients undergoing gynecological laparoscopic surgery. The method of using a pressure transducer to measure cuff pressure continuously is more convenient and accurate than a cuff pressure manometer. This trial’s methodological strengths include Internet central randomization, computer-generated assignment, allocation concealment, blinded assessment, and appropriate estimation of sample size. Considering this is a single-center study, the conclusion might not be generalizable. The result of this study could be used as a reference for future multicenter studies to confirm the effect of cuff pressure on POST and provides a scientific theoretical basis for preventing POST to further support comfort medicine.

## Trial status

This protocol (version 1.0, 16 March 2022) was approved by the Ethics Committee of Beijing Chaoyang Hospital and registered at the Chinese Clinical Trial Registry on 18 October 2022 (registration number ChiCTR2200064792). The recruitment started on 21 November 2022. When the manuscript is submitted, the enrollment is still in progress. The recruitment will be completed approximately in May 2023.

## Abbreviations

POST: Postoperative sore throat
CPMA: Cuff pressure measurement and adjustment
PONV: Postoperative nausea and vomiting
WHO: World Health Organization
VAP: Ventilator-associated pneumonia
SPIRIT: Standard Protocol Items: Recommendations for Interventional Trials
NRS: Numerical Rating Scale
BMI: Body mass index
ASA: American Society of Anesthesiologists
PASS: Power analysis and sample size
NCSS: Number Cruncher Statistical System
eCRF: Electronical case report form
SPSS: Statistical Product and Service Solutions
IBM: International business machines
